# Vet-OncoNet: Malignancy Analysis of Neoplasms in Dogs and Cats

**DOI:** 10.3390/vetsci9100535

**Published:** 2022-09-28

**Authors:** Katia Pinello, Irina Amorim, Isabel Pires, Ana Canadas-Sousa, José Catarino, Pedro Faísca, Sandra Branco, Maria C. Peleteiro, Daniela Silva, Milton Severo, João Niza-Ribeiro

**Affiliations:** 1 Vet-OncoNet, Departamento de Estudo de Populações, ICBAS, Instituto de Ciências Biomédicas Abel Salazar, Universidade do Porto, 4050-313 Porto, Portugal; 2 EPIUnit, Instituto de Saúde Pública, Universidade do Porto, 4050-600 Porto, Portugal; 3 Laboratório para a Investigação Integrativa e Translacional em Saúde Populacional (ITR), 4050-600 Porto, Portugal; 4 Departamento de Patologia e Imunologia Molecular, ICBAS, Instituto de Ciências Biomédicas Abel Salazar, Universidade do Porto, 4050-313 Porto, Portugal; 5 Departamento de Ciências Veterinárias, Universidade Trás-os-Montes e Alto Douro (UTAD), 5000-801 Vila Real, Portugal; 6 CECAV, Centro de Ciência Animal e Veterinária, Universidade de Trás-os-Montes e Alto Douro, 5000-801 Vila Real, Portugal; 7 DNAtech, Laboratório de Análises Clínicas Veterinárias, 1649-038 Lisboa, Portugal; 8 Faculdade de Medicina Veterinária, Universidade Lusófona de Humanidades e Tecnologia, 1749-024 Lisboa, Portugal; 9 CBIOS, Centro de Investigação em Biociências e Tecnologias da Saúde, Universidade Lusófona de Humanidades e Tecnologia, 1749-024 Lisboa, Portugal; 10 Instituto Mediterrâneo para a Agricultura, Ambiente e Desenvolvimento, MED, Universidade de Évora, 7006-554 Évora, Portugal; 11 VetPat®, Laboratório Veterinário, 2710-297 Lisboa, Portugal; 12 Segalab, Laboratório de Sanidade Animal e Segurança Alimentar, 4490-295 Póvoa de Varzim, Portugal

**Keywords:** cancer, cat, dog, malignancy, veterinary oncology

## Abstract

**Simple Summary:**

An overview analysis of tumors in dogs and cats, dividing them into malignant and benign, may provide previously unknown information about the biological behavior of tumors in these species and may serve many veterinarians as a support for clinical decision making. Based on a sample of 16,272 cancer records, including 3266 cats and 13,006 dogs, the analysis found that cats have a fourfold risk of malignant tumors, as in some topographies. Sex appears to play a role in the malignancy only in dogs. Some dog breeds (Pit bull and Boxer) have a higher risk of malignant tumors as opposed to Shih tzu and Yorkshire terrier. District of residence was not relevant in predicting malignancy risk. Most importantly, the risk of malignant tumors increases by 20% every three years.

**Abstract:**

Analysis of canine and feline tumor malignancy data can help clinicians identify high-risk patients and make more accurate decisions. Based on a sample of 16,272 cancer records, including 3266 cats and 13,006 dogs, collected from January 2019 to December 2021 in the Vet-OncoNet Network database, this study aimed to compare the tumor malignancy profile between cats and dogs, considering animal-related factors (sex, age, and breed), topography, and geographic location using a mixed-effects logistic regression model. Cats had a higher proportion of malignant tumors (78.7%) than dogs (46.2%), and the malignancy profile was very different regarding tumors’ topographies. The mean age of malignant tumors occurred eight months later than benign ones (9.1, SD = 3.4; 9.8, SD = 3.2), in general. Species (OR = 3.96, 95%CI 3.57: 4.39) and topography (MOR = 4.10) were the two most important determinants of malignancy risk. Female dogs had a higher risk than male dogs (OR = 1.19, 95%CI 1.08: 1.31), which does not appear to be the case in cats (OR = 0.98, 95%CI 0.77: 1.23). Breed contributed significantly to differences in malignancy risk in dogs (MOR = 1.56), particularly in pit bulls and boxers. District of residence was not so relevant in predicting malignancy risk (MOR = 1.14). In both species, the risk of malignancy increased by approximately 20% every three years. It could be hypothesized that species differences in genetic structure may contribute to tumor malignancy.

## 1. Introduction

According to recent trends [1], cancer in humans may overtake cardiovascular disease as the leading cause of premature death in most countries during this century. In developed countries, cancer is also believed to be the leading cause of disease-related deaths in the elderly [2,3,4,5,6].

It is difficult to define a tumor in absolute terms [7]. However, they can be classified into three broad types: (1) Benign tumors: they arise in any tissue of the body, grow locally, and their clinical importance lies in their ability to produce local pressure or cause obstruction [7]; (2) In situ tumors: the lesion appears to contain cancer cells, but the tumor remains within the epithelial layer, not invading the basement membrane or supporting mesenchyme; (3) Cancer: often refers to a malignant tumor characterized by uncontrolled cell division, and able to invade locally and metastasize distantly [7].

Cancer can be considered as a series of diseases (or a multistep process [7]) triggered by the accumulation of genetic mutations combined with the disruption of regulatory epigenetic mechanisms [8] that drive the progression of a normal cell into a highly malignant cancer cell [7]. The relationship between genetic mutations, proliferation, and cancer has been intensively studied in recent decades [8]. The increased risk of cancer observed in individuals occupationally exposed to mutagens, such as tobacco users, supported the multifactorial etiology of cancer [9].

Some studies have found associations between animal cancers and environmental influences [10,11,12,13,14,15], while others have shown consistent associations between certain cancers and animal breeds, suggesting heritable rather than environmental cancer risk [6,15,16,17]. Although all breeds and crossbreeds of dogs can be affected by cancer, it is noteworthy that some breeds of purebred dogs appear to be at increased risk for certain cancers, suggesting an underlying genetic predisposition to cancer susceptibility [18]. Although the etiology of most cancers is likely multifactorial, the limited genetic diversity in purebred dogs facilitates genetic linkage or association studies [18]. Most importantly, all of these cancers occur in animals past middle age, which incorporates the “aging” factor into this complex model.

In order to draw appropriate conclusions regarding cancer exposure in animals, to make recommendations for cancer prevention and control, or to design analytical studies to determine causal relationships between exposure and cancer risk, the necessary foundation are accurate cancer surveillance data [15]. The principles of epidemiology form the basis of evidence-based veterinary medicine (EBVM), an approach to health practice that is now well accepted in veterinary medicine. For clinicians, using the EBVM approach means committing to base all decision-making upon the best available evidence [15].

In a data-driven era, the concept of data analytics and business intelligence (BI) has become very important, as seen during the COVID pandemic, when public health decisions were made based on data and the resulting information [19]. BI provides a way to explore the data and allows one to understand trends and gain valuable insights into any field. In veterinary medicine, BI can provide clinicians with information that can help with decision-making and cancer management [20,21,22]. Since it is often not possible to make a diagnosis as accurately as in human medicine, information that helps clinicians predict the biological behavior of tumors based on the characteristics of the animal can be a valuable tool.

Thus, data on tumor malignancy in dogs and cats can become an important tool for predictions that can help to: (1) identify patients at high risk, (2) provide a data-based threshold for cancer screening, (3) provide information for basic research, (4) reveal species differences between cancer types, and (5) raise (or invalidate) genetic or environmental hypotheses for malignancy.

The aims of this study were to characterize malignant tumors in dogs and cats and to analyze the influence of animal-inherent factors (species, sex, age, and breed), tumor location (topography) and the external factor geographic location, on tumor malignancy.

## 2. Materials and Methods

### 2.1. Data Origin and Structure

Data of confirmed animal cancer diagnostic referred to Vet-OncoNet between January 2019 and December 2021 by six veterinary laboratory partners (VLP) in Portugal was used. VLP were DNATECH, VetPat®, Laboratory of Pathological Anatomy from the Faculty of Veterinary Medicine of the University of Lisbon, the Laboratory of Veterinary Pathology of the University of Porto, the Laboratory of Veterinary Pathology at the University of Évora, and SEGALAB. Data associated with each record included: date of diagnostic, species, age, sex, breed, clinician postal code or parish, diagnosis (morphology), as well as tumor grade and anatomical location (topography).

All the tumor cases with a valid diagnostic were included in the database; among valid records, however, some records were incomplete, given lack of information submitted by the clinical veterinarian to the VLP.

Each record represents a tumor which was classified accordingly to the anatomical location (topography) and histological type (morphology) using the Vet-ICD-O-canine-1 classification system [23], thereafter abbreviated as Vet-ICD-O. Lipomas present in the database were removed from this study. The topographies were grouped into 14 systems, as per our previous study [24]. The tumors on “Soft Tissue” represent tumors occurring in the connective, subcutaneous, and other soft tissues, when this information was available, according to Vet-ICD-O.

As for cat breeds, records with Common European, no breed, and short-haired domestic cats were all classified as “Common European”.

### 2.2. Malignancy Classification

Vet-ICD-O classifies the biological behavior of tumors within six possible categories, represented by numbers after the slash: 0 for benign tumors, 1 for tumors where there is uncertainty whether they are benign or malignant, 2 for in situ neoplasms, 3 for malignant, and 6 for the metastatic tumors [23]. A new dichotomous variable called “Malignancy” was created in the database to classify tumors according to the information sent from pathologists. Tumors coded with /0 were classified in the benign tumors category; tumors coded with /2, /3 and /6 were classified as malignant tumors; tumors coded with /1, were categorized, one by one as shown in Table 1. The criterium used was based on the grading description. Tumors classified as pre-neoplastic, or tumor-like lesions were excluded from the analysis (Table S1).

### 2.3. Statistical Analysis

Continuous data was assessed for normality using the Kolmogorov–Smirnov test. The means and standard deviations were used to describe normally distributed continuous data, and the Z-test (for proportions) and *t*-test (for mean) were calculated. Categorical data were summarized with counts and percentages, per species, sex, breed topography groups, and districts. A 95% confidence interval (95%CI) and *p*-value lower than 0.05 were considered as minimum criteria for significant differences. Analysis was performed using RStudio (version 1.4.1103). 

### 2.4. Generalized Linear Mixed Effect Models

Multilevel mixed-effects logistic regression models [30] were used, with malignancy as the outcome variable; species, age, and sex as fixed effects; and district, breed, and topography group as random effects. Both univariable and multivariable analyses used the aforementioned data structure. The likelihood ratio test was used to compare the model with and without each factor, and to evaluate the respective associations. A median odds ratio (MOR) was calculated to quantify the magnitude of variance associated with malignancy of multicategory variables (district, breed, and topography). MOR can be conceptually described as quantifying the variation between clusters (second-level variation), by comparing two individuals from two randomly selected different clusters. For example, considering two animals with the same covariates randomly selected from two different breeds, MOR is the median value of OR between the breed with the highest malignancy proportion and the breed with the lowest one. The same approach was used for district and topography. For this analysis, we first estimated a null model (model 0) containing only a random intercept without adjustment for species, age, or sex. Then, the model was adjusted for species, age, and sex (model 1) to assess the extent to which malignancy was explained by variability in the other factors. Only breeds with more than 30 records were included in this model.

## 3. Results

The study included 16,272 registries. Of these, 13,006 were from dogs (80.0%) and 3266 from cats (20.0%) (Table 1). Half of the records (52.7%) were considered malignant: in dogs the malignancy proportion (MP) was 46.2%, while in cats the MP surpassed 78% (Table 2), showing a four-fold higher chance (crude OR = 4.38; 95%CI: 3.99–4.80; *p* < 0.001) of having a malignant tumor compared to dogs. The malignancy proportions were statistically higher in females than in males from both species, but this was more significant in cats (Table 2).

Female cats showed higher odds of malignant tumors when compared to female dogs (crude OR = 4.95, 95%CI: 4.41–5.56), and the same happened with male cats compared to male dogs (crude OR = 3.31, 95%CI: 2.85–3.84).

**Figure 1:**
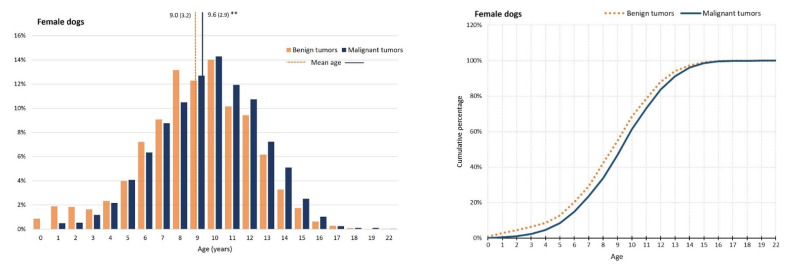

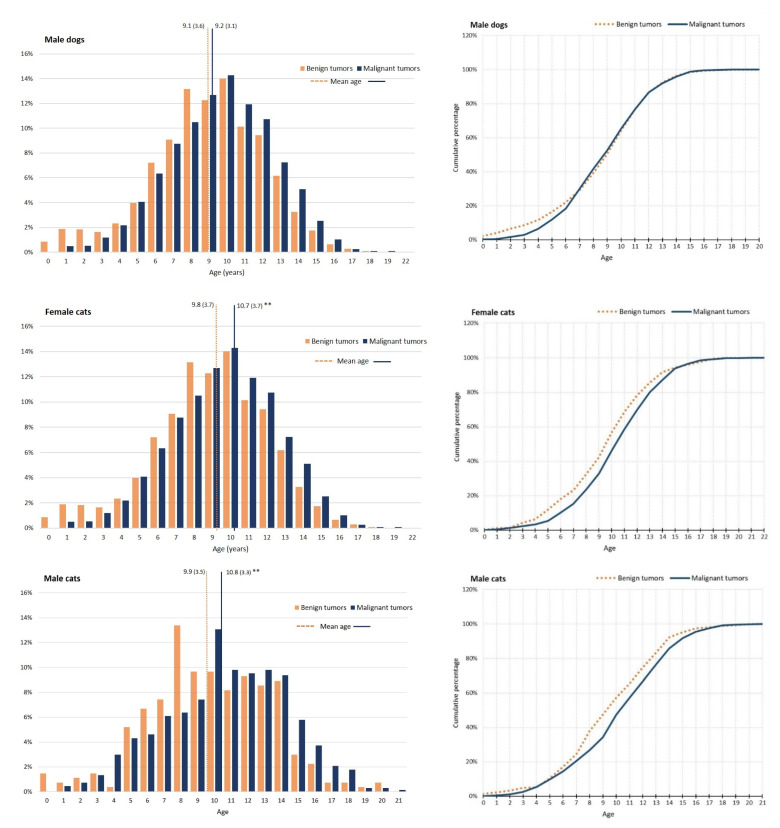
Age distribution (in percentage, (**left**)) and cumulative proportions (**right**) of benign and malignant tumors in dogs and cats, by sex. Dotted lines in the left graphics represent mean ages of benign and malignant tumors. ** *p* < 0.001, *t*-test.

Regarding age distributions, the mean age of incidence for malignant compared with benign tumors, occurred eight months later (9.1, SD = 3.4; 9.8, SD = 3.2), in general. In dogs, the mean age for malignant tumors was statistically higher than for benign ones (9.0, SD = 3.3; 9.5, SD = 3.0, *p* < 0.001), and the same tendency was observed in cats (9.9, SD = 3.5; 10.7, SD = 3.4, *p* < 0.001). This effect can be seen in Figure 1, which shows the age distribution and the correspondent cumulative incidence of malignant tumors per species and sex. In female dogs, a peak of benign tumors at 8 years old (you.) seems to be due to mammary gland benign mixed tumors and complex adenomas. In male dogs, a peak of malignant tumor at the age of 7 and 8 you. was attributable to the presence in high numbers of canine perivascular wall tumors. Additionally, the higher frequency of benign tumors in the first ages are likely caused by cases of canine cutaneous histiocytoma. In female cats, a peak of benign tumors at 10 y.o. seems to be caused by mammary gland tubular adenomas. In male cats, the peak of benign tumors reflects the presence of mast cell tumors, and the peak of malignant tumors seems to be caused by squamous cell carcinoma.

**Figure 2:**
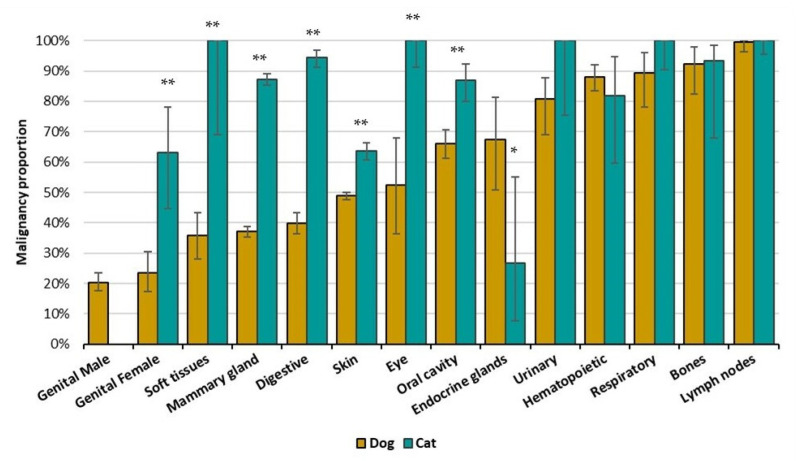
Malignancy Proportion (MP) per topography groups by species: dogs (yellow) and cats (blue). Bars indicate MP with 95%CI. Dashed line represents the 50% of malignant tumors. Differences of proportions were calculated for the species within each topography, * *p* < 0.05, ** *p* < 0.001, Z-test for proportions.

**Figure 3:**
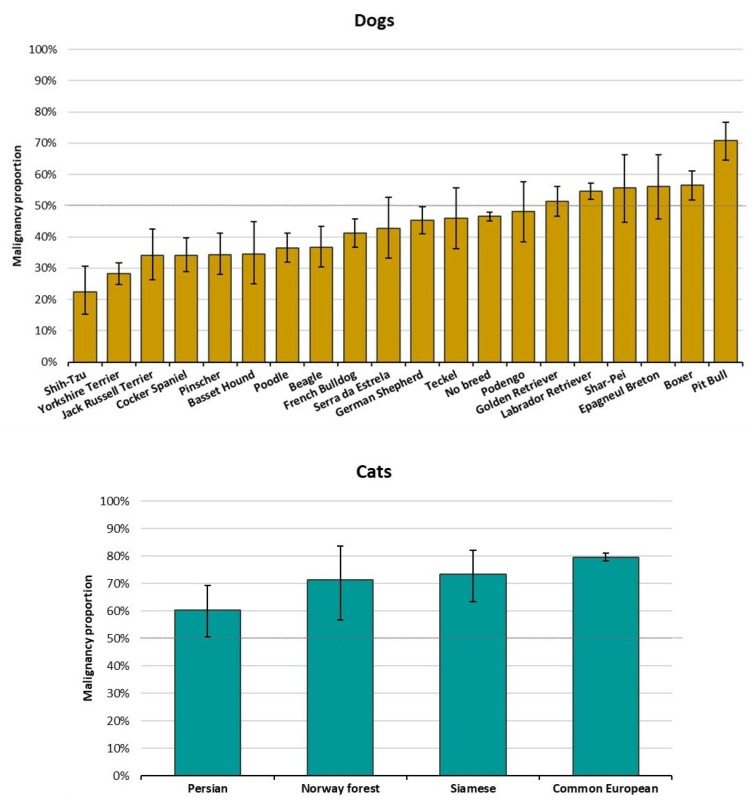
Malignancy proportion per main breeds of dogs (**top**) and cats (**down**). Bars indicate proportion of 95%CI. Dashed line depicts 50% of malignant tumors.

Regarding topographies, the three most affected systems in both dogs and cats (Tables S2 and S3) are tumors of the skin, mammary glands, and digestive organs. However, the profile of MP diverges between species, as shown in Figure 2. In dogs, 7/14 topography groups have MP higher than 50%, while in cats, only endocrine glands and genital tumors in males present a MP under 50% (Figure S1 presents data aggregated for cats only). Due to the low number of cases, the category of genital in male cats (n = 3) was removed from Figure 3. The only topography group for which cats presented a statically lower MP than dogs was the endocrine glands (*p* = 0.005).

**Figure 4:**
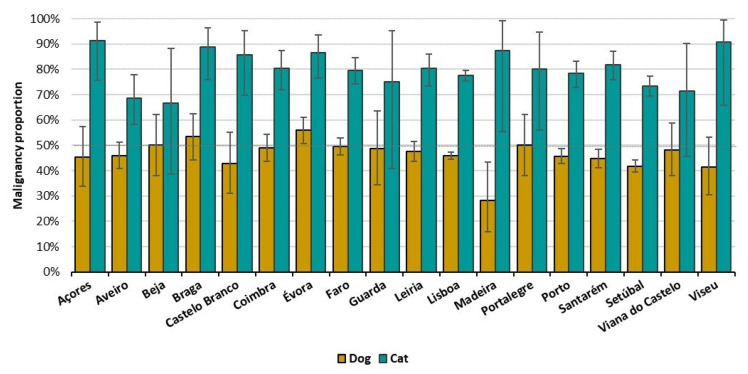
Malignancy proportion per district by species: dogs (yellow) and cats (blue). Bars indicate 95%CI. Dashed line represents 50% of malignant tumors.

Regarding breeds, the five most frequent dog breeds were no breed (n = 4850, 37.8%), Labrador retriever (n = 1445, 11.3%), Yorkshire terrier (n = 669, 5.2%), German shepherd (n = 500, 3.9%), and French bulldog (n = 444, 3.5%) (Table S4). Within the 20 most frequent breeds (Figure 4), Pit-bull and Boxer showed the higher MPs (70.9% and 56.5%, respectively) while Shih-Tzu and Yorkshire Terriers had the lowest MPs (22.2% and 28.1%, respectively).

In cats, the great majority were recorded as common European (n = 2971, 91.5%) followed by Persian (n = 103, 3.2%) (Table S5). All breeds included (n > 30) present a MP higher than 50% (Figure 3).

Regarding the geographical location, Lisbon (7536, 46.3%), Setubal (2239, 13.7%), and Porto (1400, 8.6%) are the districts with higher representation, both in dogs and cats (Tables S6 and S7). The MP was not highly different through the districts for both species (Figure 4 and Figure S2). For dogs, Madeira showed the lowest MP (28.2%) and Évora the highest one (56%) (Figure S2). For cats, all districts presented a MP higher than 50%, Beja with the lowest MP (66.7%) and Açores the highest (95.2%) (Figure 4 and Figure S2).

### Mixed Effect Logistic Regression

Table 3 shows that topography contributes the most to variation among malignancy outcome, both without adjustment (model 0) and with adjustment (model 1). The tendency and magnitude of contribution is kept four-fold in both models, and when both species are analyzed together or separately.

Breed contributes for malignancy either in dogs or cats; in dogs, the odds of malignancy varied 56% between the breed with lowest and the one with highest risk, and in cats the variability in risk was lower, at just 43%.

The contribution from districts is kept low in both models 0 or 1, and for both species analyzed together or separately; the variation in malignancy attributable to district is below 20% (11% for dogs and 19% for cats).

Species contributed to malignancy four-fold more in cats than in dogs. Age contributed with an increase in magnitude of risk of malignancy for both species (Table 3). The sex contribution to malignancy was apparent in dogs, with females showing a 19% higher risk of tumor malignancy, but not in cats, for which sex appears not to increase such risk. 

## 4. Discussion

Cancer develops through multiple molecular mechanisms underpinned by alterations at the genetic and epigenetic levels. It is important to emphasize that the pathways by which cells become malignant vary widely [7]. Mutations in specific oncogenes may occur early in some tumors and late in others. As a result, acquisition of key cancer features may occur at different times in the progression of different cancers [7].

The current study reports a conjoint analysis of malignancy for 16,272 tumors from dogs and cats submitted to Vet-OncoNet by six LabVet partners. The authors of this study believe that it is important to analyze the occurrence of tumors in dogs and cats, since spontaneous tumors in dogs and cats are an appropriate, available, and valid model system for studying cancer biology [31]. The current study explores the biologic behavior of more than 16,000 tumors, from dogs and cats in Portugal, focusing on identifying associations between possible determinants of malignancy in those tumors. An overall malignant proportion of 52.7% (46.2% in dogs and 78.7% in cats) was found, with the mean age at which malignant tumors occur generally eight months later than benign ones. The current study identified a greater risk of malignant tumors in cats vs. dogs, female vs. male dogs, in older animals, in specific breeds, and in certain topographies.

### 4.1. Species

Our results evidenced four-time higher odds of cats having a malignant tumor compared to dogs. The endocrine glands are the only topography for which dogs present more than double the cats’ MP, whereas for all the others, cats are at higher risk of malignancy.

Among the 14 topographical groups, cats showed a MP higher than 50% in 12, except for endocrine glands and genitals. Conversely, dogs’ MP was under 50% for seven topographies (Figure S1).

Cross-species variation in cancer risk shows a strong phylogenetic signal, mostly driven by cancer mortality risk in Carnivora [32]. However, cats differ from most other carnivores as a result of being obligatory carnivorous. Moreover, the number of genomic regions with strong selection signals since the domestication of the cat seems to be modest when compared to those of the domestic dog [33]. The lower odds in dogs could be genetically selected throughout the domestication and inbreeding processes. Conversely, it can also be due to selection bias caused by cancer care and consequent diagnostic in cats. However, this last hypothesis is losing strength, as the total number of the cat population is increasing. In Europe, it is estimated that the cat population is higher than dogs (110 million cats, 90 million dogs), with 26% of European households owning at least one cat, and 24% owning at least one dog [34].

### 4.2. Age

Cancer has been defined as the phenotypic end result of a whole series of changes that may have taken a long period of time to develop [7]. The age-dependent incidence of cancer suggests that between four and seven rate-limiting, stochastic events are required to produce a malignant phenotype [35,36]. In our analysis, when compared to benign ones, the malignant tumors showed a later mean age of incidence either in dogs or cats. This effect was more pronounced for cats (9 months vs. 6 months in dogs), which could be due to the longer feline lifespan.

### 4.3. Topography

Regarding the malignancy by topography groups, it is interesting to notice that only tumors of endocrine glands in cats showed a MP lower than 50%, and those tumors were the only ones with lower malignancy compared to dogs (26.7% and 67.5%, respectively). In this system, both dogs and cats showed a higher proportion of tumors in the thyroid gland (66%), followed by adrenal gland tumors (29% in dogs and 26% in cats) (data not shown). Our results tend to be similar to other reports, in which carcinomas were diagnosed in 90% of canine thyroid tumors [37,38]. In contrast, the lower MP in cats could be explained by the fact that hyperthyroidism (thyrotoxicosis) is the most common endocrine disorder [38], and it is most commonly associated with adenomatous thyroid hyperplasia, or thyroid adenomas [39].

The MP of bone and hematopoietic tissues (spleen) is similar in dogs and cats. Tumors in lymph nodes are always considered malignant due to the nature of this system.

In both species, tumors of the skin were the most frequent. In the literature, the overall incidence of tumors of the skin and subcutaneous tissues for dogs and cats is difficult to determine due to inconsistency of reporting, particularly with tumors of the subcutaneous tissues [40]. This bias could also affect our database. Only for the diagnostics that were explicit, the terms “subcutaneous” or “soft tissue” were classified as soft tissue system, if not, they were classified as skin. According to Hauch and Oblack [40] skin tumors represent 26% to 43% of all tumors (in cats 30%) and, of these, between 20% and 40% are malignant. This can be caused by the fact that lipomas were removed from our analysis. Lipomas are composed of lobulated, slow-growing, mature adipose tissue, having minimal connective tissue stroma. They are commonly enclosed in a thin, fibrous capsule [40]. Given the histological similarities with normal fat, the absence of description of capsule and in the absence of data related to the growth of the lesion, the differential diagnosis with normal fat is a challenge and could make lipomas overrepresented in our sample. Therefore, we chose not to include them in the study.

Our database shows a higher frequency of skin tumors, 49.5% for dogs and 39.6% for cats, with a lower risk of malignancy for dogs: MP of 48.4% for dogs and 62.9% for cats. The main risk factors related to skin tumors in dog and cats are UV radiation, viral infections, and immune status [40]. Evidence for the role of UV irradiation is more evidently associated with SCC (squamous cell carcinoma) development due to solar exposure of skin, in light-colored cats [40,41]. There is increasing evidence that papillomaviruses may also be a cause of cutaneous squamous cell carcinomas and basal cell carcinomas in cats [42].

Our results regarding tumors of the mammary gland are similar to other reports [18,43], which state these are the most common tumors in female dogs (50–70% of all tumor types). According to Dobson [18], the incidence of mammary tumors in dogs varies with breed, but the breeds reported to be at risk differ in different studies, and in different geographic locations. In our database, the no-breed dogs were the most affected (42%), followed by Yorkshire terrier (10%) and Poodle (6%), which is in line with previous reports [44]. These suggests that small-breed dogs, such as Poodles, Chihuahuas, Dachshunds, Yorkshire terriers, Maltese, and Cocker spaniels are the higher-risk breeds [43]. It is important to note that, in our database, the MP for mammary glands in cats was more than double the one in dogs.

Oral tumors are common in both cats and dogs, with cancers of the oral cavity accounting for 3% to 12% and 6% of all tumors in these species, respectively [45], which corroborated our findings. A recent study reported that oral tumors showed a MP of 53.6% in dogs, and 58.1% in cats [46]. Comparatively, in our data, the MP was lower in dogs, but higher in cats: 38.5% and 78.2%, respectively.

Among the tumors of digestive tract in dogs, more than 70% of tumors were located in the anus and anal canal (anal and hepatoid glands), similar to findings in other reports which state that perianal adenomas represent the majority of canine perianal tumors (58% to 96%) [45]. In our data, approximately 75% of the tumors in cats were located in the intestinal tract and 80% represented by lymphomas. A recent study stated that the incidence of all intestinal tumors reported was 0.4%, with small intestinal tumors predominating [47]. In our database, tumors in this topography are much more frequent (9.1%). 

It is common to see that oral tumors are usually analyzed within the digestive system [29,45]. However, since the tumors of the oral cavity are too heterogeneous and aggressive [45], and to allow future comparisons with human cancer registries, we treated our data separately. Both species presented a high malignancy proportion in this group being highly represented by melanomas in dogs and squamous cell carcinomas in cats.

The respiratory tract in our database is mostly represented by tumors in the nasal cavity (50.7% in dogs, and 73% in cats), which is in accordance with the literature [48,49], with a high proportion of malignant tumors [49]. In dogs, 29.2% of the tumors were from bronchus and lung (0.1% from total, data not shown). Lung cancer is the most common cause of cancer-related deaths in humans worldwide, but primary lung cancer remains relatively uncommon or underdiagnosed in domestic dogs and cats [49]. Our data corroborates the literature, with less than 1% [49].

The frequency of genital tract and mammary gland tumors may suffer from presumable bias, because a significant percentage of dogs and cats were neutered. In our database, canine genital female tract is mainly represented by tumors on the ovary (36.4%) and vagina (34.6%), while in cats there are an overrepresentation of ovarian tumors (82%), where half of the cases are adenocarcinomas (data not shown). Cats show a more than double the MP of dogs.

Regarding genital tumors of the male, our data contains only three cases of cats, of which two in the testis. This is similar to the absence in the literature reported [50]. The database contains 829 tumor cases in dogs, more than 75% of which benign tumors from testis, which is lower than usually reported [50,51]. 

The tumors on the urinary system in dogs (0.5%) are represented mostly by bladder tumors (57.6%) followed by kidney (19%), mostly carcinomas, which is in accordance with the literature [52]. In cats, these were less frequent (0.4%) and equally distributed between bladder carcinomas and kidney. However, in this last, the majority of the cases were lymphomas.

Regarding the malignancy of bone tumors, our findings corroborate previous reports. In a recent review [53], it was reported that 90% to 98% of primary bone tumors in dogs are malignant, and 92% to 95% of those in cats as well [53]. According to McNeil et al, 2007 [54], osteosarcoma of the long bones is the most common malignant tumor of the bone in dogs, accounting for 85% to 90% of primary bone tumors. It almost exclusively affects the large and giant breeds, such as Rottweiler, Great Dane, Irish wolfhound, Greyhound, and Saint Bernard [18,54]. In our database, no-breed dogs (42%), Labrador retriever (11%), Rafeiro Alentejano, and Boxer (5% each) were the breeds most affected by osteosarcoma. Our results are consistent with the hypothesis that increasing weight and body size appear to be important predictive factors for oncological disease in dogs [55].

From the detailed analysis of this section, it results that the risk of tumor malignancy is clearly higher for cats than for dogs and both species express different topography malignancy profiles.

An important note refers to the fact that not all pathologists in the group agree in considering “perianal skin” in the topographic group of the digestive system. Perianal skin falls under topography C21 (anus and anal canal) in Vet-ICD-O-canine-1. Additionally, not every author agrees to consider subcutaneous mast cell tumors as benign tumors.

### 4.4. Breed 

As stated by Dobson, 2013 [18], the existence of differences between breeds of dog and their risk of developing certain types of cancer are well recognized. However, to the authors’ knowledge, there are no aggregated analysis of malignancy across breeds. Considering only the 20 most frequent breeds in our database, the study has shown that Pitbull and Boxer have the highest MP. Both breeds present a higher frequency of mast cell tumors (data not shown). 

On the opposite side are placed Shih-Tzu and Yorkshire terrier, all small size breeds. Small breed dogs have a longer life expectancy [2] and some cancer predisposition as stated by Dobson et al. [18]. However, there are no reports regarding malignancy. Shih-Tzu are reported to have a low proportion of cancer-related death [18] and, in our database, the most common morphology was sebaceous adenoma (9.3%). Yorkshire Terrier was the second, after no-breed, in the mammary gland group; it is the breed with the highest proportion of tumors in this system (50.8%). However, the two main morphologies are complex adenoma (14%) and benign mixed tumor (10.2%).

Mainly in dogs, the increasing inbreeding over the years created an accidentally specific pool of alleles in some cases, allowing the identification of rare variants in the whole canine population [18]. In a multifactorial disease such as cancer, the impact of environmental exposure can be analyzed against a reasonably homogenous genetic background [18] either for dogs or for cats. The fact that different breeds of dog have shown different proportions of malignant behavior is not only interesting but could provide a very important insight into the necessity of studying possible cancer genetic etiology drivers [18]. In addition, the fact that, in our results, no-breed dogs with MP nearly 50% are located among pure breed with much higher MP but also much lower, reinforces the theory of the selection, in some breeds, for malignant tumors.

In cats, there are fewer genetically distinct breeds than in dogs [56,57], and our database contains only four breed categories for cats. Although according to reports, Siamese cats are overrepresented in cats with cancer in general [58], and specifically with neoplasms of the mammary gland and intestines [47], our database did not corroborate these findings, neither a particular predisposition towards malignancy in this breed. In fact, there seems to be no breed-specific significant predisposition to MP cancer contribution from different cat breeds.

### 4.5. Sex 

Sex was significantly associated with a diagnostic of a malignant tumor in this study, in a conjoint dog and cat analysis. In general, females presented 19% higher odds of having a malignant tumor, similar to female dogs. However, in cats, after multivariate analysis this chance was not confirmed, even though it appeared in the crude proportion analysis.

The cause for a sex predisposition for malignant tumors has not been reported and, due to the weak information collected regarding reproduction status in our network so far, it was difficult to set statements or raise concrete hypotheses. However, it is important to state that: (1) in general, both male and female cats are neutered more often than dogs and at younger ages; (2) there is still use of contraceptives in cats in Portugal, which can increase MP, especially in mammary tumors [32].

### 4.6. Geographical Distribution 

The contribution of geographical location to variability in tumor malignancy was assessed at the district level and revealed contribution to global variability on tumor malignancy of 14% (overall), with 11% in dogs, and 19% in cats. The results do not show that large urban districts, such as Lisbon or Porto, have the highest MP, and in less densely populated districts, such as the islands of Madeira and the Açores, there were high MP for cats but not for dogs. Interestingly, higher risk districts for malignancy in cats appear as low malignancy for dogs and the reverse is true. Our analysis focused on data with high level of aggregation, and no clear effect of geography variation at district level on malignancy appeared, after the data structure analyzed. The analysis also does not capture effects from narrower closer environmental factors, such as indoor pollution caused by various carcinogens such as tobacco, radon, and others, which may have a stronger influence on malignancy [59,60,61,62]. 

### 4.7. Limitations of the Study

One limitation comes from the diagnostic protocols followed by the different laboratories which, although having competent and experienced pathologists, do not yet follow the same standardized assay protocols. This was compensated by a thorough classification of tumor malignancy following Vet-ICD-O-canine-1. This problem runs through several studies in the field of cancer epidemiology in animals, and initiatives such as the Veterinary Cancer Guidelines and Protocols Group (vcgp.org) should be widely disseminated and adopted by pathologists.

In some regions, such as the Azores, Madeira, Beja, and Evora, there could be a selection bias by which only tumors with malignant aspect are sent to diagnostic. Factors like option for cytology, or the owner’s decision in not sending biopsies from animals with clinical diagnostic of late or terminal cancer to the lab could reduce the presence of malignant tumors. The option to not include lipomas in the analysis can influence the relations and proportions in certain topographies, especially in comparisons with other studies.

Another limitation arises from the fact that the zip codes and resulting district location used in this study belong to the veterinary clinics requesting the histopathologic examinations, not to the owners themselves. However, the fact that the geographic analysis was performed at the district level rather than the city level greatly reduced bias.

### 4.8. Important Notes

In a society where the role and active presence of pets in the family is increasing exponentially, many owners want their pets to be cared for at a similar level to themselves [15]. In order to achieve a level of care in veterinary oncology that is truly similar to that of humans, there needs to be an increased focus on the collection, standardization, active, ongoing, and consistent monitoring of cancer data. This requires increasingly integrated work. A functioning interdisciplinary working relationship between the pathologist and the clinician is essential to gain important and correct insights through data analysis. Clinicians should be increasingly involved in providing accurate information to pathologists, knowing that this information will reach epidemiologists. Building a line of information generation is the foundation to produce evidence that helps clinicians make more accurate medical decisions, creating a circle of credible information.

## 5. Conclusions

This study represents a characterization of malignancy through all types of tumors in dogs and cats. These results provide valuable information to predict the malignancy of a tumor upon animal inherent characteristics—species, breed, sex, age, and the district of residence. Species and topography were the two main determinants for the tumor risk of malignancy. Differences in the malignancy proportion were observed: felines are at higher risk than dogs and show a profound malignancy profile by topography. Female dogs are at higher risk than male dogs whereas sex does not play a role in cats’ risk of malignancy. There is a considerable contribution of breed for differences in dog risk of malignancy. Age increased the risk of malignancy for both species. District level across the country was not relevant to predict risk of malignancy. These results can be used as a hypothesis generator for future investigations.

## Figures and Tables

**Table 1:** Criteria utilized to assess the malignancy of tumors with behavior code /1.

Diagnosis	Malignancy Category
Canine cutaneous MCT [25,26] ^all grades^	Malignant
Feline cutaneous MCT [27] Without malignancy description With some malignancy description	Benign Malignant
Subcutaneous MCT [26,28] ^all pattern descriptions^	Benign
Meibomian gland epithelioma	Malignant [29]
All other /1 morphologies Without malignancy description With some malignancy description	Benign Malignant
Morphologies undefined (M800) ^#^	Not included in the analysis

MCT: mast cell tumors; ^#^ M800: Vet-ICD-O-canine group for Neoplasm, NOS (not otherwise specified).

**Table 2:** Description of the biologic behavior of tumors by species and sex.

		Total Tumors		Malignant Tumors	
Variable	Category	N	%	N	MP % ^1^
	Total	16,272	100	8578	52.7
Species					
	Canine	13,006	80.0	6008	46.2
	Feline	3266	20.0	2570	78.7 **
Sex ^2^		16,105	100	8475	52.6
	Male	6285	39.0	3123	49.7
	Female	9820	61.0	5352	54.5 **
Dog					
	Male	5275	41.0	2384	45.2
	Female	7605	59.0	3552	46.7 **
Cat					
	Male	1010	31.3	739	73.2
	Female	2215	68.7	1800	81.3 **

^1^ MP (Malignancy Proportion): Proportion of malignant tumors per respective category; ^2^ Difference between specific counts and the total count is due to missing information regarding sex; ** *p* < 0.001, Z-test for the differences between MP of categories within the same variable.

**Table 3:** Multivariable logistic regression models. Without adjusting for species, age, and sex (Model 0) and with adjustments (Model 1) for risk factors for malignant tumors in the Vet-OncoNet database. Topography of tumors (topography group), breed, and geographical location (district) were considered as random effects.

Variable	Category	Model 0		Model 1		
		OR	95%CI	OR	95%CI	*p*-Value
Dogs and cats						
Species						
	Canine	-	-	Ref		
	Feline	-	-	3.96	(3.57, 4.39)	<0.001
Age (3y)				1.18	(1.14, 1.23)	<0.001
Sex						
	Male	-	-	Ref		
	Female	-	-	1.19	(1.09, 1.30)	<0.001
**Random Effect**		**MOR**	**Variance**	**MOR**	**Variance**	
	Topography	4.26	2.314	4.10	2.188	
	District	1.09	0.009	1.14	0.019	
Dogs						
Age (3y)		-	-	1.24	(1.19, 1.29)	<0.001
Sex						
	Male	-	-	Ref		
	Female	-	-	1.19	(1.08, 1.31)	<0.001
**Random Effect**		**MOR**	**Variance**	**MOR**	**Variance**	
	Topography	4.33	2.364	4.25	2.301	
	Breed	1.53	0.198	1.56	0.221	
	District	1.09	0.009	1.11	0.013	
Cats						
Age (3y)		-	-	1.23	1.13, 1.34	<0.001
Sex		-	-			
	Male	-	-	Ref		
	Female	-	-	0.98	0.77, 1.23	0.872
**Random Effect**		**MOR**	**Variance**	**MOR**	**Variance**	
	Topography	6.44	3.811	6.05	3.563	
	Breed	1.38	0.117	1.43	0.140	
	District	1.19	0.034	1.19	0.035	

Total number of cases included in this analysis: malignant tumors: 8083, benign tumors: 7208. Only breeds with more than 30 records were included.

## Data Availability

The data presented in this study are available on request from the corresponding author. The data are not publicly available due to ethics.

## Supplementary Materials

The following supporting information can be downloaded at: https://www.mdpi.com/article/10.3390/vetsci9100535/s1, Table S1: List of morphologies classified as pre-neoplastic or tumor-like lesions, and not included in the analysis of malignancy. Figure S1: Malignancy proportion per topography group for dogs (top) and cats (down). Bars indicate 95%CI. Dashed line represents 50% of malignant tumors; Figure S2: Malignancy proportion per districts from dogs (top) and cats (down). Bars indicate 95%CI. Dashed line depicts 50% of malignant tumors; Table S2: Description of tumors from dogs according to topography groups (N, %) and proportion of malignant tumors (MP % and respective 95% Confidence Interval). Total of 13,006 records; Table S3: Description of tumors from cats according to topography groups (N, %) and proportion of malignant tumors (MP % and respective 95% Confidence Interval). Total of 3284 records; Table S4: Description of tumors from dogs according to breeds (N, %) and proportion of malignant tumors (MP % and respective 95% Confidence Interval). Total of 12,805 records; Table S5: Description of tumors from cats according to breeds (N, %) and proportion of malignant tumors (MP % and respective 95% Confidence Interval). Total of 3246 records; Table S6: Description of tumors from dogs according to districts (N, %) and proportion of malignant tumors (MP % and respective 95% Confidence Interval). Total of 12,987 records; Table S7: Description of tumors from cats according to districts (N, %) and proportion of malignant tumors (MP % and respective 95% Confidence Interval). Total of 3260 records.

## References

[1] Bray F., Laversanne M., Weiderpass E., Soerjomataram I. (2021). The ever-increasing importance of cancer as a leading cause of premature death worldwide. Cancer.

[2] Adams V.J., Evans K.M., Sampson J., Wood J.L.N. (2010). Methods and mortality results of a health survey of purebred dogs in the UK. J. Small Anim. Pract..

[3] Egenvall A., Bonnett B.N., Hedhammar Å., Olson P. (2005). Mortality in over 350,000 insured Swedish dogs from 1995–2000: II. Breed-specific age and survival patterns and relative risk for causes of death. Acta Vet. Scand..

[4] Bonnett B.N., Egenvall A., Hedhammar Å., Olson P. (2005). Mortality in over 350,000 insured Swedish dogs from 1995–2000: I. Breed-, gender-, age- and cause-specific rates. Acta Vet. Scand..

[5] Nødtvedt A., Berke O., Bonnett B.N., Brønden L. (2012). Current status of canine cancer registration—Report from an international workshop. Vet. Comp. Oncol..

[6] Sarver A.L., Makielski K.M., DePauw T.A., Schulte A.J., Modiano J.F. (2022). Increased risk of cancer in dogs and humans: A consequence of recent extension of lifespan beyond evolutionarily determined limitations?. Aging Cancer.

[7] Argyle D.J., Khanna C., Giancristofaro N., Vail D.M., Thamm D.H., Liptak J.M. (2020). 2—Tumor Biology and Metastasis. Withrow and MacEwen’s Small Animal Clinical Oncology.

[8] You J.S., Jones P.A. (2012). Cancer genetics and epigenetics: Two sides of the same coin?. Cancer Cell.

[9] Wogan G.N., Hecht S.S., Felton J.S., Conney A.H., Loeb L.A. (2004). Environmental and chemical carcinogenesis. Semin. Cancer Biol.

[10] Reif J.S. (2011). Animal sentinels for environmental and public health. Public Health Rep..

[11] Schmidt P.L. (2009). Companion animals as sentinels for public health. Vet. Clin. North Am. Small Anim. Pract..

[12] Rabinowitz P., Scotch M., Conti L. (2009). Human and animal sentinels for shared health risks. Vet. Ital..

[13] Pinello K.C., Niza-Ribeiro J., Fonseca L., de Matos A.J. (2019). Incidence, characteristics and geographical distributions of canine and human non-Hodgkin’s lymphoma in the Porto region (North West Portugal). Vet. J..

[14] Bertone E.R., Snyder L.A., Moore A.S. (2002). Environmental tobacco smoke and risk of malignant lymphoma in pet cats. Am. J. Epidemiol..

[15] Ruple A., Bonnett B.N., Page R.L., Vail D.M., Thamm D.H., Liptak J.M. (2019). 4—Epidemiology and the Evidence-Based Medicine Approach. Withrow and MacEwen’s Small Animal Clinical Oncology.

[16] Modiano J.F., Breen M., Burnett R.C., Parker H.G., Inusah S., Thomas R., Avery P.R., Lindblad-Toh K., Ostrander E.A., Cutter C.G. (2005). Distinct B-cell and T-cell lymphoproliferative disease prevalence among dog breeds indicates heritable risk. Cancer Res..

[17] Ostrander E.A., Dreger D.L., Evans J.M. (2019). Canine Cancer Genomics: Lessons for Canine and Human Health. Annu. Rev. Anim. Biosci..

[18] Dobson J.M. (2013). Breed-predispositions to cancer in pedigree dogs. ISRN Vet. Sci..

[19] Galetsi P., Katsaliaki K., Kumar S. (2022). The medical and societal impact of big data analytics and artificial intelligence applications in combating pandemics: A review focused on Covid-19. Soc. Sci. Med..

[20] Tierney A., Buchan I.E., Setzkorn C., Jones P.H., Newton J.R., Bryan J.G.E., Gaskell R.M., Coyne K.P., Noble P.J., Dawson S. (2011). SAVSNET—The Small Animal Veterinary Surveillance Network: The ’Who What Where When & Why of the UK vet-visiting companion animal population. Epidemiol. Sante Anim..

[21] Royal Veterinary College, U.o.L. and U.o. Sydney The Veterinary Companion Animal Surveillance System (VetCompass)..

[22] McGreevy P., Thomson P., Dhand N.K., Raubenheimer D., Masters S., Mansfield C.S., Baldwin T., Magalhaes R.J.S., Rand J., Hill P. (2017). VetCompass Australia: A National Big Data Collection System for Veterinary Science. Animals.

[23] Pinello K., Baldassarre V., Steiger K., Paciello O., Pires I., Laufer-Amorim R., Oevermann A., Niza-Ribeiro J., Aresu L., Rous B. (2022). Vet-ICD-O-Canine-1, a System for Coding Canine Neoplasms Based on the Human ICD-O-3.2. Cancers.

[24] Pinello K., Pires I., Castro A.F., Carvalho P.T., Santos A., de Matos A., Queiroga F., Canadas-Sousa A., Dias-Pereira P., Catarino J. (2022). Cross species analysis of tumors in dogs and cats, by age, sex, topography and main morphologies. Data from Vet-OncoNet. Vet. Sci..

[25] Willmann M., Yuzbasiyan-Gurkan V., Marconato L., Dacasto M., Hadzijusufovic E., Hermine O., Sadovnik I., Gamperl S., Schneeweiss-Gleixner M., Gleixner K.V. (2021). Proposed Diagnostic Criteria and Classification of Canine Mast Cell Neoplasms: A Consensus Proposal. Front. Vet. Sci..

[26] Bellamy E., Berlato D. (2021). Canine cutaneous and subcutaneous mast cell tumours: A review. J. Small Anim. Pract..

[27] Sabattini S., Bettini G. (2019). Grading Cutaneous Mast Cell Tumors in Cats. Vet. Pathol..

[28] Thompson J.J., Pearl D.L., Yager J.A., Best S.J., Coomber B.L., Foster R.A. (2011). Canine subcutaneous mast cell tumor: Characterization and prognostic indices. Vet. Pathol..

[29] Meuten D.J. (2017). Tumors in Domestic Animals.

[30] Larsen K., Merlo J. (2005). Appropriate assessment of neighborhood effects on individual health: Integrating random and fixed effects in multilevel logistic regression. Am. J. Epidemiol..

[31] MacEwen E.G. (1990). Spontaneous tumors in dogs and cats: Models for the study of cancer biology and treatment. Cancer Metastasis Rev..

[32] Vincze O., Colchero F., Lemaître J., Conde D.A., Pavard S., Bieuville M., Urrutia A.O., Ujvari B., Boddy A.M., Maley C.C. (2022). Cancer risk across mammals. Nature.

[33] Axelsson E., Ratnakumar A., Arendt M.-L., Maqbool K., Webster M.T., Perloski M., Liberg O., Arnemo J.M., Hedhammar Å., Lindblad-Toh K. (2013). The genomic signature of dog domestication reveals adaptation to a starch-rich diet. Nature.

[34] The European Pet Food Industry FACTS & FIGURES, 2021 Annual Report. https://europeanpetfood.org/about/statistics/.

[35] McCance K.L., Huether S.E. (2019). Pathophysiology: The Biologic Basis for Disease in Adults and Children.

[36] Vail D.M., Thamm D.H., Liptak J. (2020). Withrow and MacEwen’s Small Animal Clinical Oncology.

[37] Wucherer K.L., Wilke V. (2010). Thyroid cancer in dogs: An update based on 638 cases (1995–2005). J. Am. Anim. Hosp. Assoc..

[38] Lunn K.F., Boston S.E., Vail D.M., Thamm D.H., Liptak J.M. (2020). 26—Tumors of the Endocrine System. Withrow and MacEwen’s Small Animal Clinical Oncology.

[39] Naan E.C., Kirpensteijn J., Kooistra H.S., Peeters M.E. (2006). Results of thyroidectomy in 101 cats with hyperthyroidism. Vet. Surg..

[40] Hauck M.L., Oblak M.L., Vail D.M., Thamm D.H., Liptak J.M. (2020). 19—Tumors of the Skin and Subcutaneous Tissues. Withrow and MacEwen’s Small Animal Clinical Oncology.

[41] Murphy S. (2013). Cutaneous squamous cell carcinoma in the cat: Current understanding and treatment approaches. J. Feline Med. Surg..

[42] Munday J.S., Thomson N.A., Luff J.A. (2017). Papillomaviruses in dogs and cats. Vet. J..

[43] Moe L. (2001). Population-based incidence of mammary tumours in some dog breeds. J. Reprod. Pertil. Suppl..

[44] Sorenmo K.U., Worley D.R., Zappulli V., Vail D.M., Thamm D.H., Liptak J.M. (2019). 28—Tumors of the Mammary Gland. Withrow and MacEwen’s Small Animal Clinical Oncology.

[45] Vail D.M., Thamm D.H., Liptak J.M., Vail D.M., Thamm D.H., Liptak J.M. (2020). 23—Cancer of the Gastrointestinal Tract. Withrow and MacEwen’s Small Animal Clinical Oncology.

[46] Cray M., Selmic L.E., Ruple A. (2020). Demographics of dogs and cats with oral tumors presenting to teaching hospitals: 1996–2017. J. Vet. Sci..

[47] Rissetto K., Villamil J.A., Selting K.A., Tyler J., Henry C.J. (2011). Recent trends in feline intestinal neoplasia: An epidemiologic study of 1,129 cases in the veterinary medical database from 1964 to 2004. J. Am. Anim. Hosp. Assoc..

[48] MacEwen E.G., Withrow S.J., Patnaik A.K. (1977). Nasal tumors in the dog: Retrospective evaluation of diagnosis, prognosis, and treatment. J. Am. Vet. Med. Assoc..

[49] Vail D.M., Thamm D.H., Liptak J.M., Vail D.M., Thamm D.H., Liptak J.M. (2020). 24—Tumors of the Respiratory System. Withrow and MacEwen’s Small Animal Clinical Oncology.

[50] Lawrence J.A., Saba C.F., Vail D.M., Thamm D.H., Liptak J.M. (2019). 29—Tumors of the Male Reproductive System. Withrow and MacEwen’s Small Animal Clinical Oncology.

[51] Liao A.T., Chu P.Y., Yeh L.S., Lin C.T., Liu C.H. (2009). A 12-year retrospective study of canine testicular tumors. J. Vet. Med. Sci..

[52] Fulkerson C.M., Knapp D.W., Vail D.M., Thamm D.H., Liptak J.M. (2019). 30—Tumors of the Urinary System. Withrow and MacEwen’s Small Animal Clinical Oncology.

[53] Dittmer K.E., Pemberton S. (2021). A Holistic Approach to Bone Tumors in Dogs and Cats: Radiographic and Histologic Correlation. Vet. Pathol..

[54] McNeill C.J., Overley B., Shofer F.S., Kent M.S., Clifford C.A., Samluk M., Haney S., van Winkle T.J., Sorenmo K.U. (2007). Characterization of the biological behaviour of appendicular osteosarcoma in Rottweilers and a comparison with other breeds: A review of 258 dogs. Vet. Comp. Oncol..

[55] Ru G., Terracini B., Glickman L.T. (1998). Host related risk factors for canine osteosarcoma. Vet. J..

[56] Lipinski M.J., Froenicke L., Baysac K.C., Billings N.C., Leutenegger C.M., Levy A.M., Longeri M., Niini T., Ozpinar H., Slater M.R. (2008). The ascent of cat breeds: Genetic evaluations of breeds and worldwide random-bred populations. Genomics.

[57] Kurushima J.D., Lipinski M.J., Gandolfi B., Froenicke L., Grahn J.C., Grahn R.A., Lyons L.A. (2013). Variation of cats under domestication: Genetic assignment of domestic cats to breeds and worldwide random-bred populations. Anim. Genet..

[58] Egenvall A., Nødtvedt A., Häggström J., Ström Holst B., Möller L., Bonnett B.N. (2009). Mortality of life-insured Swedish cats during 1999–2006: Age, breed, sex, and diagnosis. J. Vet. Intern. Med..

[59] Pinello K.C., Santos M., Leite-Martins L., Niza-Ribeiro J., de Matos A.J. (2017). Immunocytochemical study of canine lymphomas and its correlation with exposure to tobacco smoke. Vet. World.

[60] Reif J.S., Dunn K., Ogilvie G.K., Harris C.K. (1992). Passive smoking and canine lung cancer risk. Am. J. Epidemiol..

[61] Zierenberg-Ripoll A., Pollard R.E., Stewart S.L., Allstadt S.D., Barrett L.E., Gillem J.M., Skorupski K.A. (2018). Association between environmental factors including second-hand smoke and primary lung cancer in dogs. J. Small Anim. Pract..

[62] Fowler B.L., Johannes C.M., O’Connor A., Collins D., Lustgarten J., Yuan C., Weishaar K., Sullivan K., Hume K.R., Mahoney J. (2020). Ecological level analysis of primary lung tumors in dogs and cats and environmental radon activity. J. Vet. Intern. Med..

