# Supplementary material for: Vet-OncoNet: Malignancy Analysis of Neoplasms in Dogs and Cats
Source: Vet Sci. 2022 Sep 28;9(10):535. doi: 10.3390/vetsci9100535 (PMC9611943; doi:10.3390/vetsci9100535)
Supplement: Supplementary file 1 [file vetsci-09-00535-s001.zip › vetsci-1919304-supplementary-after publication.pdf]

Supplementary Material

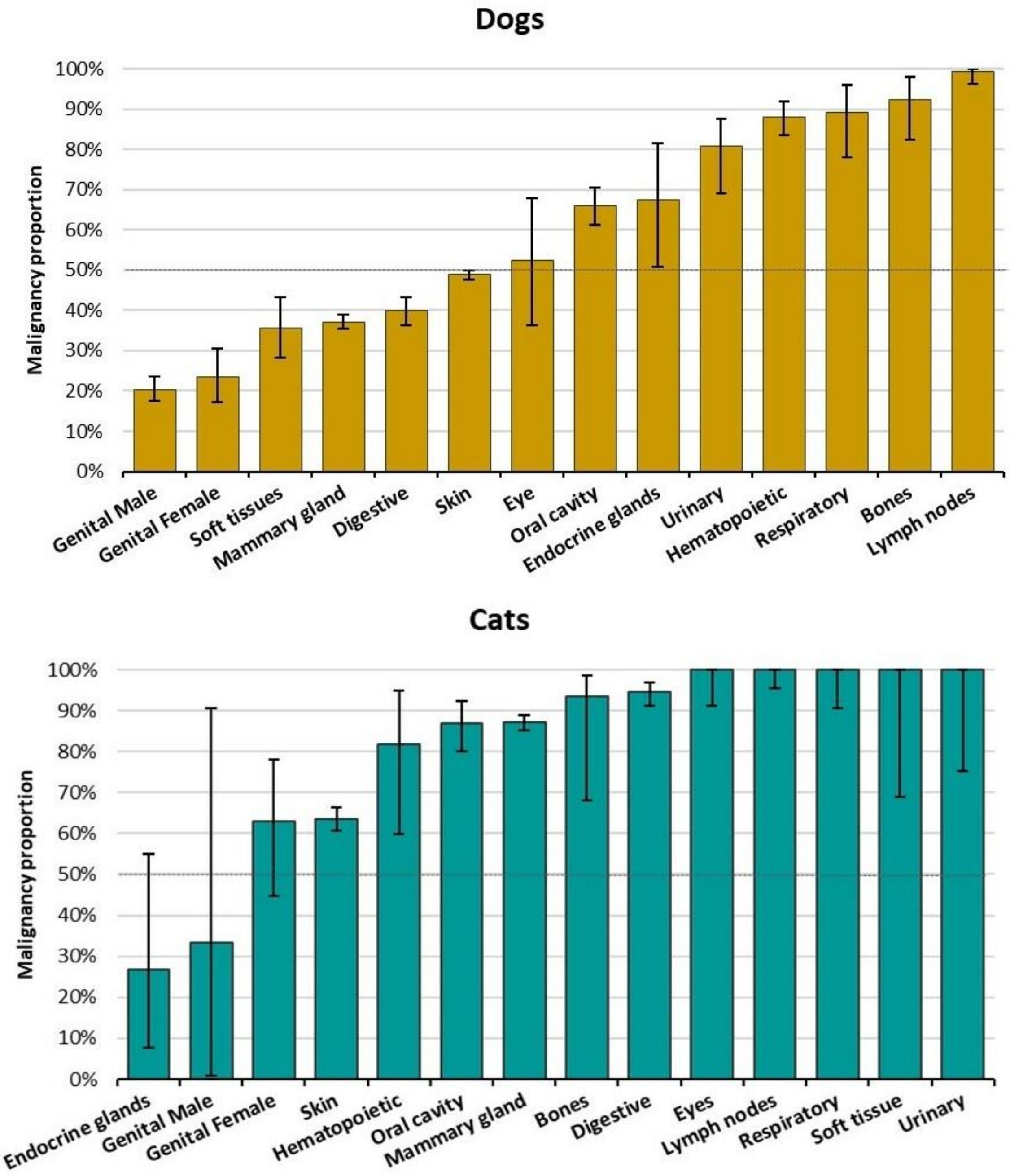

Figure S1. Malignancy proportion per topography group for dogs (top) and cats (down). Bars indicate 95% CI. Dashed line represents 50% of malignant tumours.

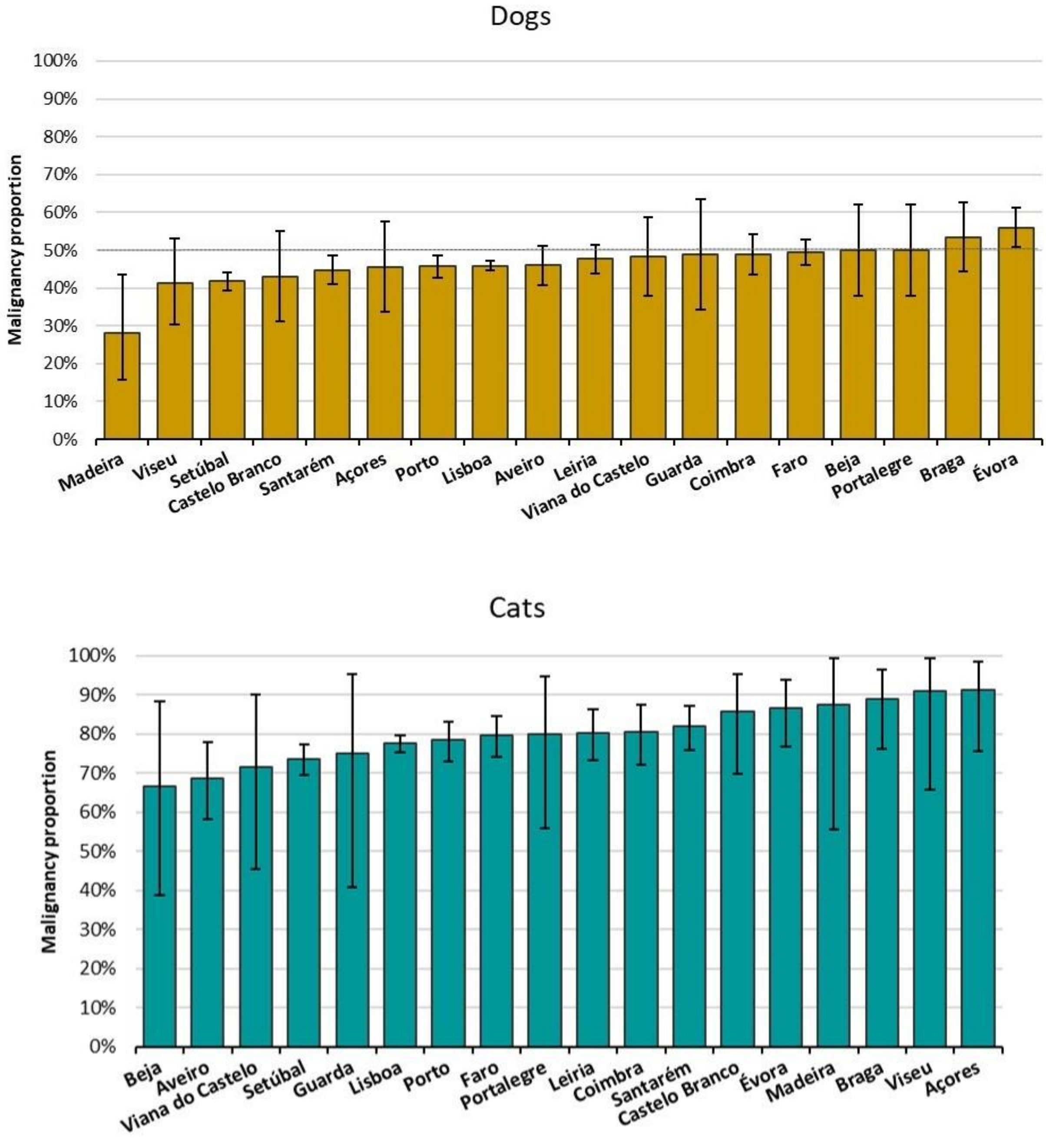

Figure S2. Malignancy proportion per districts from dogs (top) and cats (down). Bars indicate 95% CI. Dashed line depicts 50% of malignant tumours.

Table S1. List of morphologies classified as pre-neoplastic or tumour-like lesions, and not included in the analysis of malignancy.

Morphologies
Acanthomatous epulid
Acrochordon
Actinic keratosis
Adenomatous polyp
Chemodectoma
Dermoid cyst
Fibropapilloma
Hamartoma
Odontogenic fibroma
Ossifying epulis
Papillomatosis
Peripheral odontogenic fibroma
Rectal polyp

Table S2. Description of tumours from dogs according to topography groups (N, %) and proportion of malignant tumours (MP % and respective 95% Confidence Interval). Total of 13,006 records.

Topography Groups	N	%	MP %	95%CI
Skin	6421	49.5	48.8	0.475–0.500
Mammary gland	3200	24.5	37.1	0.354–0.389
Digestive	844	6.5	39.9	0.364–0.434
Genital Male	817	6.3	20.4	0.175–0.234
Oral cavity	458	3.5	66.1	0.613–0.706
Hematopoietic	278	2.1	88.0	0.835–0.917
Genital Female	193	1.5	23.5	0.173–0.306
Lymph nodes	181	1.4	99.4	0.966–1.000
Soft tissues	170	1.3	35.7	0.286–0.437
Unknown site	159	1.2	69.3	0.613–0.764
Bones and joints	67	0.5	93.0	0.829–0.980
Urinary	66	0.5	80.9	0.691–0.897
Respiratory	61	0.5	89.3	0.781–0.960
Endocrine glands	47	0.4	67.5	0.509–0.814
Eye	44	0.3	52.4	0.365–0.680
Total	13,006	100.0	46.2	0.453–0.470

Table S3. Description of tumours from cats according to topography groups (N, %) and proportion of malignant tumours (MP % and respective 95% Confidence Interval). Total of 3266 records.

Topography Groups	N	%	MP %	95%CI
Mammary gland	1270	38.9	87.0	0.852–0.889
Skin	1266	38.8	63.6	0.608–0.663
Digestive	266	8.1	94.5	0.913–0.968
Oral cavity	135	4.1	87.1	0.801–0.923
Lymph nodes	81	2.5	100.0	0.954–1.000
Unknown site	53	1.6	90.2	0.785–0.967
Eye	40	1.2	100.0	0.911–1.000
Genital Female	38	1.2	63.1	0.459–0.781
Respiratory	37	1.1	100.0	0.905–1.000
Hematopoietic	22	0.7	81.8	0.597–0.948
Bones and joints	15	0.5	93.3	0.680–0.998
Endocrine glands	15	0.4	26.7	0.078–0.551
Urinary	13	0.4	100.0	0.753–1.000
Soft tissues	12	0.4	100.0	0.615–1.000
Genital Male	3	0.1	33.3	0.008–0.906
Total	3266	100.0	78.7	0.773–0.801

Table S4. Description of tumours from **dogs** according to breeds (N, %) and proportion of malignant tumours (MP % and respective 95% Confidence Interval). Total of 12,805 records.

Breeds	N	%	MP %	95%CI
No breed	4850	37.8	46.6	0.451–0.480
Labrador Retriever	1445	11.3	54.5	0.512–0.570
Yorkshire Terrier	669	5.2	28.1	0.248–0.316
German Shepherd	500	3.9	45.3	0.409–0.496
French Bulldog	444	3.5	41.2	0.367–0.458
Boxer	430	3.7	56.5	0.518–0.611
Golden Retriever	433	3.4	51.3	0.466–0.560
Poodle	400	3.1	36.5	0.318–0.412
Cocker Spaniel	293	2.3	34.1	0.288–0.396
Pit Bull	214	1.7	70.9	0.645–0.767
Beagle	210	1.6	36.7	0.303–0.433
Pinscher	198	1.5	34.3	0.279–0.411
Jack Russell Terrier	132	1.0	34.1	0.263–0.424
Shih-Tzu	112	0.9	22.2	0.152–0.306
Podengo	102	0.8	48.0	0.384–0.577
Teckel	98	0.8	45.9	0.362–0.557
Serra da Estrela	96	0.7	42.7	0.331–0.527
Epagneul Breton	90	0.7	56.1	0.458–0.662
Basset Hound	87	0.7	34.5	0.250–0.448
Shar-Pei	79	0.6	55.7	0.447–0.663
Siberian Husky	80	0.6	50.0	0.377–0.608
Bull Terrier	78	0.6	48.7	0.378–0.597
Rafeiro Alentejano	77	0.6	58.4	0.472–0.690
Pekinese	75	0.6	40.0	0.294–0.512
Chihuahua	73	0.6	35.6	0.252–0.469
Pug	63	0.5	63.5	0.513–0.746
Rhodesian Lion	62	0.5	50.0	0.377–0.622
Cão d´água Português	56	0.4	44.6	0.320–0.576
Schnauzer	54	0.4	53.7	0.404–0.665

West Highland White Terrier	53	0.4	30.1	0.189–0.432
Dogo Argentino	51	0.4	70.5	0.572–0.818
Rottweiller	50	0.4	66.0	0.523–0.780
Weimaraner	46	0.4	45.6	0.317–0.599
Great Dane	43	0.3	53.5	0.387–0.678
German Braco	43	0.3	41.8	0.279–0.567
Spitz	41	0.3	34.1	0.209–0.493
Cane Corso	41	0.3	36.5	0.229–0.518
Portuguese pointer	39	0.3	46.1	0.311–0.616
Belgian Shepherd	38	0.3	44.7	0.296–0.605
Doberman	37	0.3	48.6	0.330–0.644
Bichon Maltese	36	0.3	41.6	0.265–0.579
Dalmatian	34	0.2	76.4	0.606–0.884
Bichon	34	0.2	23.5	0.115–0.393
Fox Terrier	30	0.2	36.5	0.210–0.544
Others breeds (n < 30)	689	5.4	48.0	0.443–0.517
Total	12,805	100.0	46.1	0.453–0.470

Table S5. Description of tumours from **cats** according to breeds (N, %) and proportion of malignant tumours (MP % and respective 95% Confidence Interval). Total of 3246 records.

Breeds	N	%	MP %	95%CI
Common European	2971	91.5	79.6	0.781–0.810
Persian	103	3.2	60.2	0.505–0.693
Siamese	83	2.6	73.5	0.633–0.821
Norway Forest	42	1.3	71.4	0.568–0.835
Others breeds (n < 30)	47	1.5	65.9	0.523–0.795
Total	3246	100.0	78.6	0.772–0.800

Table S6. Description of tumours from **dogs** according to districts (N, %) and proportion of malignant tumours (MP % and respective 95% Confidence Interval). Total of 12,987 records.

Districts	N	%	MP %	95%CI
Lisboa	5933	45.6	45.9	0.445–0.472
Setúbal	1765	13.6	41.8	0.394–0.441
Porto	1162	9.0	45.7	0.427–0.486
Faro	951	7.3	49.4	0.462–0.527
Santarém	712	5.5	44.7	0.410–0.484
Leiria	677	5.2	47.6	0.438–0.515
Évora	402	3.1	56.0	0.507–0.611
Aveiro	370	2.8	45.9	0.407–0.512
Coimbra	341	2.6	48.9	0.435–0.543
Braga	125	1.0	53.5	0.443–0.625
Viana do Castelo	95	0.7	48.3	0.379–0.587
Beja	80	0.6	50.0	0.379–0.620
Açores	79	0.6	45.4	0.337–0.574
Viseu	71	0.6	41.4	0.303–0.531
Portalegre	72	0.6	50.0	0.379–0.620
Castelo Branco	67	0.5	48.2	0.379–0.587
Guarda	46	0.4	48.8	0.343–0.635
Madeira	39	0.3	28.2	0.157–0.434
Total	12,987	100.0	46.2	0.443–0.460

Table S7. Description of tumours from **cats** according to districts (N, %) and proportion of malignant tumours (MP % and respective 95% Confidence Interval). Total of 3260 records.

**Districts**	**N**	**%**	**MP %**	**95%CI**
Lisboa	1603	48.9	78.2	0.761–0.801
Setúbal	474	14.5	74.5	0.705–0.783
Porto	238	7.3	80.1	0.747–0.849
Faro	213	6.5	81.6	0.759–0.865
Santarém	182	5.6	82.7	0.768–0.878
Leiria	150	4.6	80.6	0.737–0.865
Coimbra	104	3.2	80.8	0.723–0.877
Aveiro	79	2.4	70.5	0.598–0.798
Évora	61	1.9	86.7	0.766–0.937
Braga	37	1.1	88.9	0.760–0.964
Castelo Branco	28	0.9	85.7	0.699–0.953
Açores	22	0.7	95.2	0.806–0.997
Viana do Castelo	16	0.5	71.4	0.455–0.901
Portalegre	14	0.4	85.7	0.620–0.974
Beja	12	0.4	66.7	0.387–0.882
Viseu	11	0.3	90.9	0.657–0.995
Guarda	8	0.2	75.0	0.408–0.953
Madeira	8	0.2	85.7	0.506–0.991
Total	3260	100.0	78.7	0.773–0.801
